# Child and mother study satisfaction in a longitudinal study of children at-risk for type 1 diabetes

**DOI:** 10.1186/s12887-026-07251-1

**Published:** 2026-07-02

**Authors:** Jessica Melin, Roy Tamura, Holly O’Donnell, Suzanne Bennett Johnson

**Affiliations:** 1 Department of Clinical Science Malmö, Lund University, Box 50332, CRC 60 Pl 11, Malmö, 202 13 Sweden; 2 Health Informatics Institute, University of South Florida, Tampa, FL USA; 3 Department of Pediatrics, Barbara Davids Center for Diabetes, University of Colorado School of Medicine, Aurora, CO USA; 4 Department of Behavioral Sciences and Social Medicine, Florida State University College of Medicine, Tallahassee, FL USA

**Keywords:** Study satisfaction, Child, Parent, Genetic risk, Type 1 diabetes, Longitudinal study

## Abstract

**Background:**

Child study satisfaction has rarely been assessed in longitudinal pediatric research. This study examined child and maternal study satisfaction among 10–15-year-old participants in The Environmental Determinants of Diabetes in the Young (TEDDY) study, an observational multinational (US, Finland, Germany, and Sweden) study of children at increased genetic risk for type 1 diabetes enrolled at birth.

**Methods:**

Child and maternal study satisfaction was measured by a 3-item annual questionnaire from child-age 10 years until the last visit at child-age 15. Generalized estimating equations identified sociodemographic and study-related variables associated with study satisfaction.

**Results:**

Children and mothers exhibited high study satisfaction across all ages although their study satisfaction scores were weakly correlated (r ≥ 0.20, *p* < 0.001). Factors associated with higher study satisfaction were similar for children and mothers: living in the US compared to Finland (Finnish children: -0.78 95%CI -0.86, -0.69; Finnish mothers: -1.48 95%CI -1.61, -1.35; *p* < 0.001), older child age (15-year-old vs 10-year-old child: 0.09 95%CI 0.03,0.14; mother: 0.42 95%CI 0.37, 0.47; *p* < 0.001), and accurate perception about the child’s type 1 diabetes risk (child: 0.20 95%CI 0.15,0.25; mothers: 0.20 95%CI 0.13, 0.22; *p* < 0.001). Additional factors associated with greater child study satisfaction included female sex (-0.29 95%CI -0.35, -0.24; *p* < 0.001), being multiple islet autoantibody positive (0.21 95%CI 0.11, 0.32; *p* < 0.001), lower child anxiety about type 1 diabetes risk (-0.01 95%CI -0.02, -0.01; *p* < 0.001), having a mother with higher study satisfaction (0.13 95%CI 0.12, 0.15; *p* < 0.001), and having read an age-appropriate book about the study, its purpose, and type 1 diabetes (0.19 95%CI 0.12, 0.25; *p* < 0.001).

**Conclusions:**

Study satisfaction was high among 10–15-year-old children at increased genetic risk for type 1 diabetes, and their mothers, who had participated in a longitudinal observational study since the child’s birth. Factors associated with satisfaction were similar for children and their mothers. Potentially modifiable factors that could be targeted to improve study satisfaction include mother and child accuracy about the child’s type 1 diabetes risk and for child study satisfaction in particular – the child’s own anxiety about type 1 diabetes, the child’s mother’s satisfaction and having the child read high quality age-appropriate materials about the study.

**Trial registration:**

NCT00279318, 06/09/2004.

**Supplementary Information:**

The online version contains supplementary material available at 10.1186/s12887-026-07251-1.

## Background

Although participants’ satisfaction with a research protocol is important from both an ethical and a practical perspective, longitudinal studies focusing on pediatric populations have rarely assessed participant study satisfaction. Those that have usually collected data from parents at the end of the study. Crane and colleagues reviewed the child study satisfaction literature and found the majority of children had a positive attitude toward research participation; they wanted to learn something new, help others, and many were willing to participate in another study. Few of the reviewed publications reported negative effects of study participation, such as anxiety, being bored, or worrying about being identified as high risk for a disease [1]. However, most studies investigated the children’s perspective at the end of the study and no study investigated factors associated with child study satisfaction [1].

A few type 1 diabetes screening studies have collected information on parent and child perspectives on study participation. In All Babies in the Southeast of Sweden (ABIS), a longitudinal study following children at increased risk for type 1 diabetes, over 80% of mothers reported a positive attitude toward study participation [2] and the majority of ABIS children were satisfied with the information they were provided about the study although factors associated with their satisfaction were not assessed [3].

The Diabetes Prevention Trial for Type 1 Diabetes (DPT-1) measured overall study satisfaction among adult participants, parents of participating children, and child participants ≥ 10 years of age at the end of the study; parents were more likely to be satisfied with their participation than their children and the adult participants. However, no factors related to study satisfaction were investigated [4]. The Environmental Determinants of Islet Autoimmunity (ENDIA) study, a pregnancy-birth cohort study of high-risk children in Australia, reported study satisfaction data from a subset of caregivers and children who completed a survey (51% of eligible caregivers and 38% of eligible children); over 50% of the children were happy or very happy with their overall participation and 95% of the caregivers indicated that their participation was an excellent or good experience. Parents with a negative attitude were more likely to be inactive in the study. Caregiver and child ratings were significantly correlated [5].

The Environmental Determinants of Diabetes in the Young (TEDDY) study is an international, longitudinal study to identify environmental triggers of islet autoimmunity and type 1 diabetes among high-risk children. Children were screened for increased genetic risk for type 1 diabetes at birth and followed until 15 years of age [6]. Parent study satisfaction was collected annually from study inception and has been found to be high among both mothers and fathers. Factors such as accurate perception of the child’s risk for type 1 diabetes, belief that something can be done to reduce the child’s risk, low parent education, and meeting the same staff at most of the study visits were found to be associated with greater parent study satisfaction [7]. Parent study satisfaction has also been found to be important for both TEDDY study retention and protocol compliance [8–10]. Mothers dissatisfied with their participation were more likely to withdraw from TEDDY [8]. Greater parent study satisfaction measured in the first year of TEDDY was found to be associated with greater study visit compliance in the following 3 years of this longitudinal study [10] and more satisfied mothers were more likely to bring their multiple islet autoantibody positive child to the TEDDY clinic for the Oral Glucose Tolerance Test [9]. Beginning at age 10, TEDDY children are asked about their own satisfaction with participation in TEDDY.

## Method

### Aim

The aims of this investigation were: (a) to examine TEDDY children’s overall study satisfaction starting at child age 10 and ending at the last TEDDY visit when the child is 15, (b) to identify factors associated with child study satisfaction, and (c) to examine the relationship between child study satisfaction and maternal study satisfaction, as well as similarities and differences between factors associated with child study satisfaction and maternal study satisfaction.

### The TEDDY study

Between 2004 and 2010, children with high-risk type 1 diabetes-related genes (selected HLA DR-DQ genotypes) were identified at birth in four different countries (Finland, Germany, Sweden and US), enrolled (*n* 8676) at the age of 3–4.5 months, then followed with study visits 2–4 times per year until they developed type 1 diabetes or until they reached 15 years of age. The protocol consisted of structured interviews, questionnaires, food records, and biological samples including a venous blood draw analyzed for islet autoantibodies. Information about well-being, risk perception, type 1 diabetes related anxiety, and study satisfaction were collected annually by questionnaire from both mothers and fathers separately and from the TEDDY children from the age of 10 years. All participating countries’ ethical boards approved the study and all of the participants’ parents consented to participate in the study [6].

### Type 1 diabetes risk communication/education in TEDDY

The TEDDY study risk communication protocol has been previously described in depth [11, 12]. In brief, parents were informed of their child’s increased genetic risk for type 1 diabetes at the time of enrollment; this information was repeated after every study visit. At enrollment and for the remaining of the study, parents of children who did not develop any islet autoantibody were told that 3 out of 100 children will develop type 1 diabetes before the age of 15, and 7 out of 100 during their lifetime. Should the child develop a single persistent autoantibody, parents were told their child’s risk had increased and that 15 out of 100 children with a single persistent autoantibody will go on to develop type 1 diabetes. Should the child develop two or more persistent autoantibodies, the parent was informed that the child’s risk had increased; more specifically, 70 out of 100 children with similar autoantibody results will develop type 1 diabetes within 10 years.

Extensive age-appropriate educational materials were developed by the study coordinators represented from all TEDDY sites, together with a psychologist, for use with the children themselves [12, 13]. Before their 10-year study visit, children were given the Will and Emma Meet the TEDDY Scientists book which provided additional information about the study to prepare them to answer their own annual questionnaire. For the children who had difficulty reading the book, parts of the book were available as short videos. In the child’s first questionnaire at age 10 they were asked: “Did you read the book, Will and Emma Meet the TEDDY Scientists: No = No- I got the book, but I did not read it or No- I did not get the book, Yes = Yes- I read part of the book or Yes- I read all of the book”.

### Study sample/population

In the TEDDY study, 5536 children completed at least one visit between age 10 to 15. This investigation included all children (*n* = 5018, 91%) and all mothers (*n* = 4874, 88%) with at least one study satisfaction measure from the annual questionnaire completed between child age 10 to 15 years. Mothers were selected as the target parent for analysis because father data was often missing and mothers were the predominant caretaker participating in TEDDY study visits.

## Study satisfaction

### Child study satisfaction

Consistent with the study satisfaction measure used in the DPT-1 [4, 14], child TEDDY study satisfaction was measured by three questions on the annual questionnaire given to the child starting at age 10: 1) “How do you feel about being in the TEDDY study? (scored 2 = I like it a lot, 1 = It is ok, 0 = I do not like it at all)”, 2) “How do you feel about your parents’ decision that you should be in TEDDY? (scored 2 = It was a good decision, 1 = It was an okay decision, 0 = It was a bad decision)” and 3) “If you had a friend who was asked to be in a study like TEDDY would you tell them they should do it? (scored 2 = Yes, 1 = Maybe, 0 = No)”. The three items were summed to a study satisfaction score with a range between 0 and 6, in which higher scores indicate higher study satisfaction. Children with 2 or 3 items missing were excluded. If 1 item was missing (1.1% of all satisfactions measures), it was replaced by the mean of the remaining two items. If an imputed satisfaction score resulted in a non-integer, the score was rounded to the nearest integer. Coefficient alpha reliability estimates were acceptable, ≥ 0.71 for all ages except age 10 (α = 0.66). Children completed their first questionnaire at child-age 10 at the clinic. They were supposed to do it independently and if they needed help, the TEDDY staff helped the children to read and explain the questions. Annual questionnaires between 11–15 years of age were completed online through a secure TEDDY portal or by paper form.

### Maternal study satisfaction

Consistent with the study satisfaction measure used in the DPT-1 [4, 14], maternal study satisfaction was measured by questionnaire from child-age 6 and 15 months and annually thereafter with three questions: 1) “Overall, how do you feel about having your child participate in the TEDDY study? (scored 2 = like it a lot, 1 = like it a little, 0 = it is ok, dislike it a little or dislike it a lot),” 2) “Do you think your child’s participation in TEDDY was a good decision? (scored: 2 = a great decision, 1 = a good decision, 0 = an ok decision, a bad decision or a very bad decision)’” and 3) “Would you recommend the TEDDY study to a friend? (scored: 2 = yes, 1 = maybe, 0 = no).” The three items were summed to a study satisfaction score with a range between 0 and 6, where higher scores indicate higher study satisfaction. In the current study, we used maternal study satisfaction scores from the annual questionnaires completed at child-age 10 to 15. Any study satisfaction measure with 2 or 3 items missing was excluded; if 1 item was missing (8.7% of all satisfaction measures), it was replaced by the mean of the remaining two items. If an imputed satisfaction score resulted in a non-integer, the score was rounded to the nearest integer. Coefficient alpha reliability estimates for mothers were acceptable, ≥ 0.70 at all child ages. Annual questionnaires were completed online through a secure TEDDY portal or by paper form.

## Factors tested for association with study satisfaction

### Sociodemographic measures

Sociodemographic variables included were country (Finland, Germany, Sweden, and US), child sex, child has a first-degree relative (mother, father, or sibling) with type 1 diabetes (yes/no), mothers age at child’s birth, parent’s first child (yes/no), and child’s ethnic minority status (for US children: if the mother was not born in US, the mother’s first language is not English, or the child is a member of an ethnic minority group; for European children: country of birth or mother’s first language is other than of the TEDDY country in which the child is living). Information about parents’ marital status (living together yes/no) and parents education level (three different categories; primary education (high school or less), trade school or some college, and graduated college/university or higher) was collected and updated periodically throughout the study. This analysis used the most recent information available as of child-age 10.5 years.

### Long distance protocol

Children not living near a TEDDY clinic could participate through a long-distance protocol. In most of these cases, the families completed interviews over the phone, questionnaires via a secure TEDDY portal, and the blood draw was performed remotely and sent to the TEDDY clinic. For this analysis, it is categorized as yes/no.

### Child islet autoantibody status

Blood drawn at each study visit was analyzed for four different type 1 diabetes-related islet autoantibodies (insulin, glutamic acid decarboxylase, insulinoma-associated protein 2, and zinc transporter 8). As children’s risk for developing type 1 diabetes increased with increasing numbers of islet autoantibodies [15], islet autoantibody status was categorized into three groups; negative (the child had not developed any islet autoantibodies for type 1 diabetes), single positive (the child had developed one persistent islet autoantibody), and multiple positive (the child had developed two or more persistent islet autoantibodies for type 1 diabetes).

### Child risk perception

The child’s perception about his/her own risk of developing type 1 diabetes was collected on the annual questionnaire, starting at child-age 10 years and continuing until the child aged out of the study at 15 years old. Children responded to the following question: “Risk is the chance that something may or may not happen. What do you think about your risk of getting diabetes: a smaller risk of getting diabetes than my friends who are not in TEDDY, the same risk of getting diabetes as my friends who are not in TEDDY, a higher risk of getting diabetes than my friends who are not in TEDDY, or I am not sure about my risk of getting diabetes”. Responses were categorized into three groups: 1) Not sure, 2) Accurate (higher risk of getting diabetes than my friends) and 3) Inaccurate (smaller or same risk as my friends).

### Maternal risk perception

Maternal risk perception was measured annually with the following question: “Compared to other children, do you think your child’s risk for developing diabetes is: much lower, somewhat lower, about the same, somewhat higher, or much higher. Responses of “higher” or “much higher” were categorized as accurate, while all other responses were categorized as inaccurate. For the purposes of this study, mothers’ responses to this question on the annual questionnaires completed at child age 10 through 15 were used.

### Child anxiety

The child’s anxiety about his/her own risk of developing type 1 diabetes was measured on the annual questionnaire starting at age 10 using the 6-item short form (SAI-CH) [16] of the State Anxiety component of the State-Trait Anxiety Inventory for Children [17]. The children were asked to think about their own risk of developing type 1 diabetes when responding to the 6 items. The items were combined and converted to the 20-item scale score [16]. Scores range from 20–80 with higher scores indicating higher anxiety.

### Maternal anxiety

Maternal anxiety about their child’s risk of developing type 1 diabetes was measured on the annual questionnaire using the 6-item short form (SAI) [18] of the State Anxiety component of the State- Trait Anxiety Inventory for adults [19]. The 6-item short form score was converted to the 20-item scale score with a range between 20–80; higher scores indicate higher anxiety [18]. For the purposes of this study, mothers’ SAI scores from the annual questionnaires completed at child age 10 through 15 were used.

### Data analysis

Child and maternal study satisfaction scores were calculated by child age for the total study sample and by country. Correlations between mother and child study satisfaction scores were calculated by child age for all child/mother pairs. Dependent t-tests examined possible differences between mother and child study satisfaction scores by country and age.

Factors associated with children’s and mothers’ study satisfaction were analyzed by a generalized estimating equation (GEE). Analysis assuming ordered multinomial distribution, a cumulative logit link function and an independent working correlation matrix. A forward stepwise procedure was used to determine the fixed subject covariates of the analysis. Candidate fixed covariates were 1) country, 2) sex, 3) ethnic minority, 4) first born child, 5) first-degree relative, 6) mother’s education, 7) long distance protocol, 8) maternal age at child’s birth, and 9) parents living together. Child age (10, 11, 12, 13, 14, 15) was always included as a categorical covariate in the model. Candidate covariates were included using the alpha = 0.05 cut-off. Once a covariate was included in the model, it was not removed in the stepwise procedure.

After the fixed subject covariates were determined, child islet autoantibody status followed by a forward stepwise procedure of two other time varying covariates was used to determine whether any of these three time-varying covariates were associated with child (or mother) study satisfaction. In addition to islet autoantibody status, the two other time varying covariates were 1) child (or mother) risk perception, and 2) child (or mother) anxiety. Time varying covariates also used the alpha = 0.05 cut-off. In the model examining covariates of child study satisfaction, mother’s study satisfaction was also included as a time varying factor. The final models only included variables with a *p*-value < 0.05 in either the child or mother study satisfaction model.

Because of missing data regarding whether the children read the educational book, this variable was added to the final child study satisfaction model in a secondary analysis to examine whether reading the educational book was associated with child study satisfaction.

Given the large sample size, *p* values of < 0.001 are emphasized here.

## Results

The demographic characteristics of the study cohort at the time that the child completed his/her first study satisfaction measure is provided in Table 1. The distribution between female and male children was equal (female 49%) and the majority of the children had no family member with type 1 diabetes (87%). Ethnic minority participants were rare (14%), and few participated on a long-distance protocol (15%). At the time of the child’s first study satisfaction assessment, only 9% of the children had developed one or multiple islet autoantibodies.

**Table 1:** Demographics of child satisfaction cohort at the time of first study satisfaction questionnaire completion

Variable	*n* (%) or mean (SD) (*n* = 5018)
Country	
United States	1997 (40)
Finland	1141 (23)
Germany	275 (5)
Sweden	1605 (32)
Gender	
Female	2469 (49)
Male	2549 (51)
First-Degree Relative	
No	4380 (87)
Yes	638 (13)
Ethnic Minority	
No	4283 (86)
Yes	684 (14)
First born child	
No	4358 (87)
Yes	649 (13)
Parents live together	
No	887 (18)
Yes	4120 (82)
Mothers Education	
Primary	668 (13)
Some college	1117 (22)
College degree	3220 (64)
Mothers age at childbirth	31.0 (5.0)
Long Distance Protocol	
No	4242 (85)
Yes	776 (15)
Islet autoantibody status	
None	4573 (91)
Single positive	251 (5)
Multiple positive	194 (4)

### Child study satisfaction

**Fig. 1:**
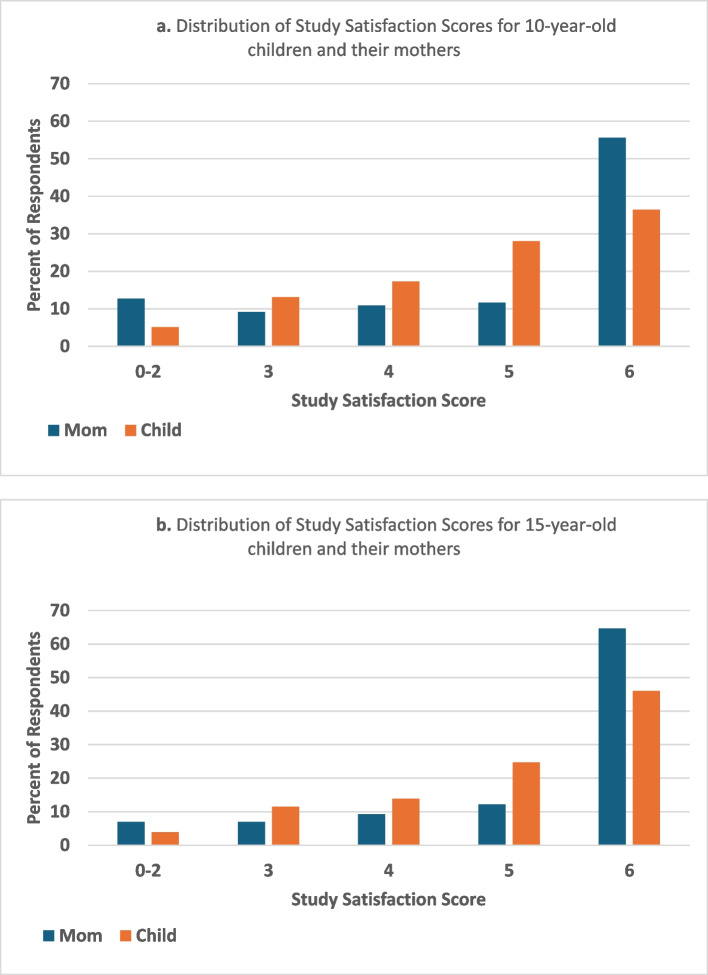
**a **Distribution of study satisfaction scores for 10-year-old children and their mothers. **b** Distribution of Study Satisfaction Scores for 15-year-old children and their mothers

Child study satisfaction scores ranged between 0 and 6, where a score of 6 is the highest study satisfaction score possible. Responses were highly skewed toward higher satisfaction with 36% of 10-year-old children and 46% of the 15-year-old participants having the highest possible score (see Fig. 1a and b). Table 2 indicates that the mean child study satisfaction score was relatively stable across age with the highest mean score at 15 years of age. Child study satisfaction scores were weakly related to mother study satisfaction scores at each child age from 10 to 15, ranging from r = 0.20 to r = 0.28 (*p* < 0.001).

**Table 2:** Child and mother study satisfaction for the overall cohort and each country by age of child

	10 y old	11y old	12y old	13y old	14y old	15y old
	mean (SD^a^, SK^b^)	mean (SD^a^, SK^b^)	mean (SD^a^, SK^b^)	mean (SD^a^, SK^b^)	mean (SD^a^, SK^b^)	mean(SD^a^, SK^b^)
Child Satisfaction:	*n*: 3738	*n*: 3933	*n*: 4005	*n*: 3939	*n*: 3896	*n*: 3406
Overall	4.7 (1.3, −1.0)	4.7 (1.3, −0.9)	4.6 (1.4, −0.8)	4.6 (1.4, −0.9)	4.7 (1.4, −0.9)	5.0 (1.2, −1.2)
United States	4.9 (1.2, −1.1)	4.9 (1.2, −1.0)	4.9 (1.2, −1.1)	4.9 (1.2, −1.2)	4.9 (1.2, −1.1)	5.2 (1.3, −1.6)
Finland	4.1 (1.4, −0.5)	4.0 (1.4, −0.4)	4.0 (1.3, −0.2)	3.9 (1.3, −0.3)	3.8 (1.3, −0.2)	4.5 (1.3, −0.7)
Germany	4.9 (1.3, −1.3)	4.9 (1.1, −0.9)	4.9 (1.3, −0.8)	4.7 (1.3, −1.0)	4.8 (1.3, −0.7)	4.9 (1.2, −1.0)
Sweden	5.0 (1.1, −1.3)	5.0 (1.2, −1.3)	4.9 (1.3, −1.2)	4.9 (1.3, −1.3)	5.0 (1.3, −1.3)	5.1 (1.2, −1.3)
Mother Satisfaction:	*n*: 3409	*n*: 3671	*n*: 3718	*n*: 3618	*n*: 3569	*n*: 3098
Overall	4.8 (1.7, −1.1)	4.9 (1.6, −1.2)	4.9 (1.6, −1.3)	4.9 (1.6, −1.3)	5.0 (1.5, −1.4)	5.2 (1.4, −1.7)
United States	5.3 (1.3, −1.8)	5.3 (1.3, −1.9)	5.3 (1.2, −2.0)	5.4 (1.2, −2.0)	5.4 (1.1, −2.2)	5.5 (1.0, −2.4)
Finland	3.7 (1.9, −0.3)	3.8 (1.9, −0.2)	3.8 (1.9, −0.2)	3.9 (1.9, −0.3)	4.1 (1.8, −0.3)	4.4 (1.8, −0.7)
Germany	4.5 (1.6, −0.8)	4.6 (1.7, −0.9)	4.7 (1.6, −0.9)	4.7 (1.5, −0.8)	4.8 (1.5, −1.2)	4.9 (1.5, −1.2)
Sweden	4.9 (1.5, −1.3)	5.1 (1.4, −1.5)	5.2 (1.3, −1.6)	5.2 (1.3, −1.7)	5.2 (1.3, −1.8)	5.4 (1.1, −2.1)

^a^Standard Deviation

^b^Skewness

Table 3 describes all factors significantly associated with child study satisfaction. Finnish children were less satisfied with the study compared to US children (*p* < 0.001) and boys were less satisfied than girls (*p* < 0.001). Age of the child was important; 11–14-years old were slightly less satisfied than 10-year-olds and 15-year-olds were the most satisfied of all (*p* < 0.001). Children who were more anxious about their type 1 diabetes risk were less satisfied (*p* < 0.001). Children who developed multiple islet autoantibodies (*p* < 0.001) and children with an accurate perception of their own risk for type 1 diabetes (*p* < 0.001) had higher study satisfaction scores compared to those who never developed any islet autoantibodies and those who were inaccurate about their type 1 diabetes risk. Children who were unsure of their risk were the least satisfied of all (*p* < 0.001). Child study satisfaction was significantly associated with their mother’s study satisfaction (*p* < 0.001).

**Table 3:** Generalized estimating equation model for child study satisfaction (*n* = 4848)

Variable	Estimate	95% CI	*p*-value
Country			
United States	Reference		< 0.001
Finland	−0.78	−0.86, −0.69	
Germany	0.08	−0.06, 0.21	
Sweden	−0.02	−0.09, 0.05	
Age of the child			< 0.001
10 years	Reference		
11 years	−0.10	−0.14, −0.05	
12 years	−0.16	−0.21, −0.12	
13 years	−0.19	−0.24, −0.14	
14 years	−0.18	−0.23, −0.13	
15 years	0.09	0.03, 0.14	
Gender			< 0.001
Female	Reference		
Male	−0.29	−0.35, −0.24	
Mothers’ education			0.004
Primary	Reference		
Some college	−0.12	−0.21, −0.02	
College degree	−0.14	−0.22, −0.06	
First born child			0.038
No	Reference		
Yes	0.09	0.00, 0.17	
Long Distance Protocol			0.038
No	Reference		
Yes	−0.10	−0.19, −0.01	
Islet autoantibody status			< 0.001
None	Reference		
Single positive	0.05	−0.06, 0.17	
Multiple positive	0.21	0.11, 0.32	
Child risk perception			< 0.001
Inaccurate	Reference		
Unsure	−0.15	−0.20, −0.09	
Accurate	0.20	0.15, 0.25	
Mothers study satisfaction	0.13	0.12, 0.15	< 0.001
Child anxiety (SAI)	−0.01	−0.02, −0.01	< 0.001

In a secondary analysis, we examined whether reading the educational Will and Emma book given to the child at 10 years of age was associated with child study satisfaction in the subset of children (*n* = 3661) for whom we had this variable. When added to the full model in Table 3, children who reported reading the book also reported greater study satisfaction (0.19, 95% CI 0.12, 0.25, *p* < 0.001). All other variables in the model remained significant.

### Maternal study satisfaction

Maternal study satisfaction scores ranged between 0 and 6, where a score of 6 is the highest study satisfaction score possible. Maternal study satisfaction scores were highly skewed toward greater study satisfaction with 54% of mothers of 10-year-old children and 64% of the mothers of 15-year-old participants having the highest possible scores (see Fig. 1a and b). Table 2 suggests some country differences, with Finnish mothers having consistently lower scores. Maternal study satisfaction also appeared to increase with increasing child age.

Table 4 describes all factors significantly associated with maternal study satisfaction. Compared to US mothers, mothers of all European countries reported lower study satisfaction (*p* < 0.001). Child age was also important, with a linear positive association between increasing child age and increasing maternal study satisfaction (*p* < 0.001). Mothers with a college degree were less satisfied than those with a high school education or less (*p* < 0.001) but those participating with their first-born child were more satisfied (*p* < 0.001). Mothers with an accurate perception of their child’s risk for type 1 diabetes had higher study satisfaction scores compared to those who underestimated the child’s risk for the disease (*p* < 0.001).

**Table 4:** Generalized estimating equation model for maternal study satisfaction (*n* = 4839)

Variable	Estimate	95% CI	*p*-value
Country			
United States	Reference		< 0.001
Finland	−1.48	−1.60, −1.35	
Germany	−0.71	−0.93, −0.50	
Sweden	−0.24	−0.32, −0.15	
Age of the child			< 0.001
10 years	Reference		
11 years	0.09	0.05, 0.13	
12 years	0.13	0.09, 0.17	
13 years	0.17	0.13, 0.22	
14 years	0.25	0.20, 0.29	
15 years	0.42	0.37, 0.47	
Gender			0.539
Female	Reference		
Male	−0.02	−0.10, 0.05	
Mothers’ education			< 0.001
Primary	Reference		
Some college	−0.03	−0.16, 0.10	
College degree	−0.30	−0.41, −0.18	
First born child			< 0.001
No	Reference		
Yes	0.21	0.10, 0.32	
Long Distance Protocol			0.040
No	Reference		
Yes	−0.13	−0.24, −0.01	
Islet autoantibody status			0.931
None	Reference		
Single positive	0.02	−0.14, 0.18	
Multiple positive	0.03	−0.16, 0.22	
Mothers risk perception			< 0.001
Inaccurate	Reference		
Accurate	0.20	0.13, 0.22	
Mother’s anxiety (SAI)	−0.004	−0.007, −0.000	0.046

## Discussion

Similar to previous publications regarding children’s and mothers’ attitudes to research participation [1–5], the majority of 10–15-year-old TEDDY children and their mothers reported high satisfaction with their overall study participation, receiving the highest possible score of 5 or 6 on the study satisfaction measure used in this study.

Both the DPT-1 trial and the ENDIA study reported similar results. In the ENDIA study, parents’ and children’s experience of their participation was significantly correlated [5]. Our analysis showed similar results; children were more likely to be satisfied with their participation if their mothers were satisfied. Similar patterns have been reported previously in TEDDY for both type 1 diabetes related anxiety and risk perception. TEDDY children’s anxiety scores when thinking of their own risk for type 1 diabetes correlated with their mothers’ anxiety scores about the child’s risk for the disease [16]. Children whose mothers had an accurate perception of their child’s type 1 diabetes risk were more likely to be accurate about their own type 1 diabetes risk [12].

Although child and mother study satisfaction scores were correlated, the association was weak (r = 0.20 to 0.28 across child age 10–15) which may be explained by the fact that it was not the child’s decision to participate, it was the parents who enrolled the child at birth. Another reason could be the skewed distribution and limited range of the satisfaction scores that may have attenuated the correlation. However, the relatively weak association between child and mother study satisfaction scores highlights the importance of assessing satisfaction directly from the children rather than relying on parents’ reports.

Although factors associated with parent study satisfaction have been previously reported in TEDDY [7], no previous publications have investigated factors associated with children’s overall study satisfaction. We found factors associated with study satisfaction were very similar between children aged 10–15 and their mothers. Clear country differences emerged for both children and mothers. Participants from the US were more satisfied than participants from other TEDDY countries. This could be a function of cultural differences, differences in familiarity with type 1 diabetes between countries, or country differences in volunteering for research studies. Although all children received birthday gifts, US children received money or gift cards after some visits which was not allowed by the research review board in the European countries. This might have contributed to the higher study satisfaction ratings of US participants. However, questionnaire completion rates could have also played a role. While differences in study satisfaction between the US and Finland were particularly notable, Finnish participants had the highest questionnaire completion rate at the last study visit; 89% of the Finnish children and 83% of the Finnish mothers complete the questionnaire compared to 67% of the US children and 65% of their mothers. This could have affected the results if participants who did not complete the questionnaire in the US failed to do so because they were less satisfied with the study.

We found that girls were more likely to be satisfied with their study participation compared to boys at the same age. Previous publications have described differences between girls’ and boys’ experiences in research participation. In the ABIS study, girls (10–13 years) had a more positive attitude toward type 1 diabetes screening and were more likely to want to know their risk of the disease compared to boys in the same age group [3, 20]. In TEDDY and in the DPT-1 trial, mothers had higher satisfaction scores than fathers, suggesting that there are differences between sexes [4, 7]. However, we did not find any associations between mother’s study satisfaction and the sex of the child participating in the TEDDY study.

The age of the child was important for overall study satisfaction among the children and even more important among their mothers, with the highest mean satisfaction scores for both children and mothers on the last visit at child-age 15 years. This might be explained by the fact that the protocol became less demanding after age 10 (e.g., food records were no longer required and accelerometry data were collected only in islet autoantibody positive children) or the fact that the 15-year-old visit was the final visit, feelings of gratitude but also a relief that the study is ending could have affect the scores. At age 10, children were first given the Will and Emma book – the most comprehensive explanation of the purpose of the TEDDY study and their own risk for type 1 diabetes up to that point in the study- which may have influenced their study satisfaction. Previous studies have shown that less satisfied mothers were more likely to withdraw from the study [8]; those who stayed in the study until 15 years of age may have been those most satisfied with the experience.

The child’s islet autoantibody status was associated with child study satisfaction but not mother study satisfaction. Children’s satisfaction increased with the number of islet autoantibodies and their increased risk of developing type 1 diabetes. Children developing islet autoantibodies during the study were seen more frequently and received more information about the disease. These children likely realized the importance of monitoring symptoms and disease progression; study staff and study participation may have also offered important reassurance.

Children and mothers with accurate perceptions of the child’s type 1 diabetes risk were more likely to be satisfied with participation in the study, which is in line with the results from the previous study of parent satisfaction [7]. Those with accurate perceptions about type 1 diabetes risk may have appreciated the importance of the TEDDY study with associated higher study satisfaction. It is noteworthy that children who were unsure about their risk of type 1 diabetes were less satisfied than children who underestimated their risk for the disease or children who were accurate about their risk. Children who were unsure about their risk may have experienced greater confusion about the study and hence lower study satisfaction. At 10 years of age, 32% of TEDDY children were unsure about their risk compared to 10% at the last 15-year visit. The greater knowledge and understanding associated with increasing age may have reduced the number of children unsure of their risk with an associated increase in positive feelings about study participation. It is important to note that accurate perception of type 1 diabetes risk has been shown to be associated with increased anxiety in both children and parents in the TEDDY study [16, 21].

We found that anxiety about type 1 diabetes risk was associated with lower study satisfaction in children and to a weaker extent in mothers. Study participation may have provided families with reassurance that staff were watching the child for signs and symptoms of type 1 diabetes.

Children who read the Will and Emma educational book received before the child age 10 study visit were more likely to be satisfied with their participation in the following years. The education material provided in TEDDY has also been associated with child risk perception accuracy; 10-year-old participants who read the book were more likely to be accurate about their risk of developing type 1 diabetes compared to those who did not read the book [12]. Previous research has shown that children want to receive information, both oral and written about their study participation and want to be involved in the decision to be part of the study [3, 22]. Education and information about the aim of the study, type 1 diabetes, and risk for the disease does not only increase the children’s overall knowledge about their study participation, but it also increases positive attitudes toward research participation.

Some studies have reported significant associations between the child’s ethnic-minority status and study recruitment [11, 23, 24] and parent anxiety about the child’s type 1 diabetes risk [24]. Other studies have reported significant associations between whether or not the child has a first-degree relative with type 1 diabetes and study retention [8, 11, 21, 24, 25]. However, we did not find child or maternal study satisfaction to be associated with either the child’s ethnic minority status or the child’s first-degree relative type 1 diabetes status. This is consistent with our prior study documenting no association between the child’s ethnic minority status or first-degree type 1 diabetes relative status and TEDDY maternal study satisfaction assessed earlier in the study (child age 15 months and 4 years) [7].

We recognize that findings from this select group of children may not generalize to other pediatric populations, including younger children, children followed for shorter periods of time, children with no risk for type 1 diabetes or who face different health challenges. A possible study limitation and bias may be study drop-out among those less satisfied with the study.

Nevertheless, our findings suggest several ways that study satisfaction might be enhanced, which in turn could reduce study drop-out and increase study compliance. Although demographic factors such as country, child sex, child age or parent education are not modifiable, several factors associated with study satisfaction in children and adults are clearly modifiable. These include mother and child accuracy about the child’s type 1 diabetes risk, whether the child has read high quality age-appropriate educational materials about the study, and child anxiety about the child’s type 1 diabetes risk, and for the child – the child’s mother’s own study satisfaction. All of these would be useful areas to target and test interventions to improve child and mother’s experiences in longitudinal pediatric studies.

It is also reassuring that children with one or more type 1 diabetes islet autoantibodies reported very high levels of study satisfaction despite their increased risk for clinical disease. Their study satisfaction scores were significantly higher than their counterparts who remained islet autoantibody negative throughout the 15-year study. This suggests that the TEDDY study was helpful to these very high-risk children and they valued their participation.

## Conclusions

Study satisfaction was high among 10–15-year-old children and their mothers who had participated in TEDDY, the longitudinal observational study of children at genetic risk for type 1 diabetes since birth. Factors associated with higher study satisfaction were similar for children and mothers: living in the US, older child age, and accurate perception about the child’s type 1 diabetes risk. Additional factors that were associated with greater child satisfaction included female sex, being islet autoantibody positive, child anxiety about their own type 1 diabetes risk, having a mother with higher study satisfaction, and having read an age-appropriate book about the study, its purpose and type 1 diabetes. Modifiable factors such as accurate perception about type 1 diabetes risk, anxiety about one’s own diabetes risk, and high-quality age-appropriate educational materials may be important targets for future study efforts to increase children and their mothers’ satisfaction with study participation.

## Supplementary Information

Supplementary Material 1.

## Data Availability

Data from The Environmental Determinants of Diabetes in the Young (10.58020/y3jk- × 087) reported here will be made available for request at the NIDDK Central Repository (NIDDK-CR) website, Resources for Research (R4R), https://repository.niddk.nih.gov/.

## Abbreviations

ABIS: All Babies in the Southeast of Sweden
DPT-1: The Diabetes Prediction Trial for Type 1 Diabetes
ENDIA: The Environmental Determinants of Islet Autoimmunity study
GEE: Generalized Estimating Equation
SAI: State Anxiety Inventory for adults
SAI-CH: State Anxiety Inventory for children
TEDDY: The Environmental Determinants of Diabetes in Young
