# Supplementary material for: Child and mother study satisfaction in a longitudinal study of children at-risk for type 1 diabetes
Source: BMC Pediatr. 2026 Jul 2;26:622. doi: 10.1186/s12887-026-07251-1 (PMC13335234; doi:10.1186/s12887-026-07251-1)
Supplement: Supplementary file 1 — Supplementary Material 1. [file 12887_2026_7251_MOESM1_ESM.docx]

Additional file 1

**The TEDDY Study Group**

**Colorado Clinical Center:** Marian Rewers, M.D., Ph.D., PI^1,4,6,9,10^, Kimberly Bautista^11^, Judith Baxter^8,911^, Daniel Felipe-Morales, Brigitte I. Frohnert, M.D., Ph.D.^2,13^, Marisa Stahl, M.D.^12^, Patricia Gesualdo^2,6,11,13^, Michelle Hoffman^11,12,13^, Randi Johnson, Ph.D.^2,3^, Rachel Karban^11^, Edwin Liu, M.D.^12^, Jill Norris, Ph.D.^2,3,11^, Holly O’Donnell, Ph.D.^8^, Andrea Steck, M.D.^3,13^, Kathleen Waugh^6,7,11^. University of Colorado, Anschutz Medical Campus, Barbara Davis Center for Childhood Diabetes, Aurora, CO, USA.

**Finland Clinical Center:** Jorma Toppari, M.D., Ph.D., PI^¥^1,4,10,13^, Olli G. Simell, M.D., Ph.D., Annika Adamsson, Ph.D.^^11^, Suvi Ahonen*^±§^, Mari Åkerlund*^±§^, Sirpa Anttila^µ¤^, Leena Hakola, Ph.D.*^±^, Sanni Heikura^µ¤^, Tiia Honkanen^µ¤^, Heikki Hyöty, M.D., Ph.D.*^±6^, Jorma Ilonen, M.D., Ph.D.^¥3^, Saori Itoshima, M.D.^¥^^, Sanna Jokipuu^^^, Taru Karjalainen^µ¤^, Leena Karlsson^^^, Jukka Kero, M.D., Ph.D.^¥^3, 13^, Marika Korpela^µ¤^, Jaakko J. Koskenniemi M.D., Ph.D.^¥^^, Miia Kähönen^µ¤11,13^, Mikael Knip, M.D., Ph.D.*^±^, Minna-Liisa Koivikko^µ¤^, Katja Kokkonen*^±^, Merja Koskinen*^±^, Mirva Koreasalo*^±§2^, Kalle Kurppa, M.D., Ph.D.*^±12^, Salla Kuusela, M.D. ^µ¤^, Jutta Laiho, Ph.D.*^6^, Tiina Latva-aho^µ¤^, Laura Leppänen^^^, Katri Lindfors, Ph.D.*^12^, Maria Lönnrot, M.D., Ph.D.*^±6^, Elina Mäntymäki^^^, Markus Mattila, Ph.D.*^±2^, Maija E. Miettinen, Ph.D.^§2^, Teija Mykkänen^µ¤^, Tiina Niininen^±^*^11^, Sari Niinistö, Ph.D.^§2^, Noora Nurminen^*±^, Sami Oikarinen, Ph.D.*^±6^, Hanna-Leena Oinas*^±^, Paula Ollikainen^µ¤^, Zhian Othmani^¥^, Sirpa Pohjola ^µ¤^, Jenna Rautanen^§^, Mia Rein^µ¤^, Minna Romo^^^, Juulia Rönkä^µ¤^, Nelli Rönkä^µ¤^, Noora Ruotsalainen^µ¤^, Satu Simell, M.D., Ph.D.^¥12^, Päivi Tossavainen, M.D.^µ¤^, Mari Vähä-Mäkilä^¥^, Eeva Varjonen^^11^, Riitta Veijola, M.D., Ph.D.^µ¤13^, Irene Viinikangas^µ¤^, Suvi M. Virtanen, M.D., Ph.D.*^±§2^. ^¥^University of Turku, Turku, Finland, *Tampere University, Tampere, Finland, ^µ^University of Oulu, Oulu, Finland, ^^^Turku University Hospital, Wellbeing Services County of Southwest Finland, Turku, Finland, ^±^Tampere University Hospital, Wellbeing Services County of Pirkanmaa, Tampere, Finland, ^¤^Oulu University Hospital, Wellbeing Services County of North Ostrobothia, Oulu, Finland, ^§^Finnish Institute for Health and Welfare, Helsinki, Finland.

**Georgia/Florida Clinical Center:** Richard McIndoe, Ph.D., PI^^4,10^, Desmond Schatz*, M.D.*^4,7,8^, Diane Hopkins^^11^, Michael Haller, M.D.*^13^, Melissa Gardiner^^11^, Ashok Sharma^^^, Ph.D.^^^, Laura Jacobsen, M.D.*^13^, Percy Gordon^^^, Jennifer Hosford*. ^^^Center for Biotechnology and Genomic Medicine, Augusta University, Augusta, GA, USA. *University of Florida, Pediatric Endocrinology, Gainesville, FL, USA.

**Germany Clinical Center:** Anette G. Ziegler, M.D., PI^1,3,4,10^, Ezio Bonifacio Ph.D.*, Cigdem Sanverdi, Anja Heublein, Sandra Hummel, Ph.D.^2^, Annette Knopff^7^, Melanie Köger, Sibylle Koletzko, M.D.^¶12^, Claudia Ramminger^11^, Roswith Roth, Ph.D.^8^, Jennifer Schmidt, Marlon Scholz, Joanna Stock^8,11,13^, Katharina Warncke, M.D.^13^, Lorena Müller, Christiane Winkler, Ph.D.^2,11^. Forschergruppe Diabetes e.V. and Institute of Diabetes Research, Helmholtz Zentrum München, Forschergruppe Diabetes, and Klinikum rechts der Isar, Technische Universität München, Neuherberg, Germany. *Center for Regenerative Therapies, TU Dresden, Dresden, Germany, ^¶^Dr. von Hauner Children’s Hospital, Department of Gastroenterology, Ludwig Maximillians University Munich, Munich, Germany.

**Sweden Clinical Center:** Åke Lernmark, Ph.D., PI^1,3,4,5,6,8,9,10^, Daniel Agardh, M.D., Ph.D.^6,12^, Carin Andrén Aronsson, Ph.D.^2,11,12^, Rasmus Bennet, Malin Goldman Tsubarah, Emelie Ericson-Hallström, Lina Fransson, Berglind Jonsdottir, M.D., Ph.D.^11^, Naghmeh Karimi, Helena Elding Larsson, M.D., Ph.D.^6,13^, Markus Lundgren, M.D., Ph.D.^13^, Jessica Melin, Ph.D.^11^, Kobra Rahmati, Anita Ramelius, Falastin Salami, Ph.D., Evelyn Tekum Amboh, Carina Törn, Ph.D.^3^. *Past staff: Eva Andersson, Corrado Cilio, Ph.D., M.D., Marie Andersson Turpeinen, Rawya Antar, Maria Ask, Jenny Bremer, Sylvia Bianconi Svensson, Ulla-Marie Carlsson, Susanne Dahlberg, Magdalena Delikat Kulinski, Annika Fors, Ulla Fält, Thomas Gard, Joanna Gerardsson, Emina Halilovic, Monika Hansen, Anna Hansson, Carina Hansson, Gertie Hansson, Susanne Hyberg, Elin M. Hård af Segerstad, Ph.D., Hanna Jisser, Fredrik Johansen, Linda Jonsson, Silvija Jovic, Sigrid Lenrick Forss, Barbro Lernmark, Ph.D., Marielle Lindström, Maria Markan, Theodosia Massadakis, Marlena Maziarz, Ph.D., Zeliha Mestan, Maria Månsson Martinez, Caroline Nilsson, Emma Nilsson, Yohanna Nordh, Karin Ottosson, Sara Rang, Anna Rosenquist, Monika Sedig Järvirova, Sara Sibthorpe, Anette Sjöberg, Birgitta Sjöberg, Ulrika Swartling Ph.D., Erika Trulsson, Ulrika Ulvenhag, Anne Wallin, Ingrid Wigheden, Terese Wiktorsson, Åsa Wimar, Sofie Åberg.* Lund University, Lund, Sweden.

**Washington Clinical Center:** William A. Hagopian, M.D., Ph.D., PI^^1,3,4,6,7,10,12,13^, Michael Killian*^6,7,11,12^, Claire Cowen Crouch*^11,13^, Jennifer Skidmore*^2^, Ben Kim*, Cody McCall*, Arlene Meyer*, Jared Radtke*, Shreya Roy*. ^^^Indiana University, Indianapolis, IN, USA. *Pacific Northwest Research Institute, Seattle, WA, USA.

**Pennsylvania Satellite Center:** Dorothy Becker, M.D., Margaret Franciscus, MaryEllen Dalmagro-Elias Smith^2^, Ashi Daftary, M.D., Mary Beth Klein, Chrystal Yates. Children’s Hospital of Pittsburgh of UPMC, Pittsburgh, PA, USA.

**Data Coordinating Center:** Jeffrey P. Krischer, Ph.D., PI^1,4,5,9,10^, Rajesh Adusumali, Sarah Austin-Gonzalez, Maryouri Avendano, Brant Burkhardt, Ph.D.^6^, Martha Butterworth^2^, Nicholas Cadigan, Joanna Clasen, Ph.D., Kevin Counts, Laura Gandolfo, Jennifer Garmeson, Veena Gowda, Shu Liu, Kristian Lynch, Ph.D. ^6,8^, Jamie Malloy, Lazarus Mramba, Ph.D.^2^, Cristina McCarthy^11^, Hemang M. Parikh, Ph.D.^3,8^, Cassandra Remedios, Chris Shaffer, Susan Smith^11^, Noah Sulman, Ph.D., Dena Tewey, Henri Thuma, Michael Toth, Ulla Uusitalo, Ph.D.^2^, Kendra Vehik, Ph.D.^4,5,6,8,13^, Ponni Vijayakandipan, Melissa Wroble, Jimin Yang, Ph.D., R.D.^2^, Kenneth Young, Ph.D. *Past staff: Michael Abbondondolo, Lori Ballard, Sandra Baethke, Rasheedah Brown, David Cuthbertson, Stephen Dankyi, Christopher Eberhard, Steven Fiske, David Hadley, Ph.D., Kathleen Heyman, Belinda Hsiao, Christina Karges, Francisco Perez Laras, Hye-Seung Lee, Ph.D., Qian Li, Ph.D., Xiang Liu, Ph.D., Colleen Maguire, Wendy McLeod, Aubrie Merrell, Steven Meulemans, Jose Moreno, Ryan Quigley, Laura Smith, Ph.D., Roy Tamura, Ph.D.* University of South Florida, Tampa, FL, USA.

**Project scientist:** Beena Akolkar, Ph.D.^1,3,4,5,6,7,9,10^. National Institutes of Diabetes and Digestive and Kidney Diseases, Bethesda, MD, USA.

**Other contributors:** Thomas Briese, Ph.D.^6^, Columbia University, New York, NY, USA. Todd Brusko, Ph.D.^5^, University of Florida, Gainesville, FL, USA. Teresa Buckner, Ph.D.^2^, University of Northern Colorado, Greeley, CO, USA. Suzanne Bennett Johnson, Ph.D.^8,11^, Florida State University, Tallahassee, FL, USA. Eoin McKinney, Ph.D.^5^, University of Cambridge, Cambridge, UK. Tomi Pastinen, M.D., Ph.D.^5,6^, The Children’s Mercy Hospital, Kansas City, MO, USA. Steffen Ullitz Thorsen, M.D., Ph.D.^2^, Department of Clinical Immunology, University of Copenhagen, Copenhagen, Denmark, and Department of Pediatrics and Adolescents, Copenhagen University Hospital, Herlev, Denmark. Eric Triplett, Ph.D.^6^, University of Florida, Gainesville, FL, USA.

***Committees:***

^1^Ancillary Studies, ^2^Diet, ^3^Genetics, ^4^Human Subjects/Publicity/Publications, ^5^Immune Markers, ^6^Infectious Agents, ^7^Laboratory Implementation, ^8^Psychosocial, ^9^Quality Assurance, ^10^Steering, ^11^Study Coordinators, ^12^Celiac Disease, ^13^Clinical Implementation.
